# Distribution and Characteristics of Bacteria Isolated from Cystic Fibrosis Patients with Pulmonary Exacerbation

**DOI:** 10.1155/2022/5831139

**Published:** 2022-12-24

**Authors:** Soroor Erfanimanesh, Mohammad Emaneini, Mohammad Reza Modaresi, Mohammad Mehdi Feizabadi, Shahnaz Halimi, Reza Beigverdi, Vajiheh Sadat Nikbin, Fereshteh Jabalameli

**Affiliations:** ^1^Department of Microbiology, School of Medicine, Tehran University of Medical Sciences, Tehran, Iran; ^2^Medical Mycology and Bacteriology Research Center, Kerman University of Medical Sciences, Kerman, Iran; ^3^Pediatric Pulmonary Disease and Sleep Medicine Research Center, Children's Medical Center, Tehran University of Medical Sciences, Tehran, Iran; ^4^Cystic Fibrosis Research Center, Iran CF Foundation (ICFF), Tehran, Iran; ^5^Department of Microbiology, Pasteur Institute of Iran, Tehran, Iran; ^6^Research Center for Antibiotic Stewardship and Antimicrobial Resistance, Tehran University of Medical Sciences, Tehran, Iran

## Abstract

**Background:**

Cystic fibrosis (CF) is an inherited recessive disorder characterized by recurrent and persistent pulmonary infections, resulting in lung function deterioration and early mortality.

**Methods:**

A cross-sectional study was conducted on the bacterial profile and antibiotic resistance pattern of 103 respiratory specimens from CF patients with signs of pulmonary exacerbation. Antibiotic susceptibility testing and biofilm formation of *Staphylococcus aureus* and *Pseudomonas aeruginosa* isolates were performed by the Kirby–Bauer disc diffusion method and microtiter plate assay, respectively. Molecular typing of *S. aureus* and *P. aeruginosa* isolates was carried out by spa typing and repetitive extragenic palindromic element PCR.

**Results:**

In a total of 129 isolates, the most prevalent organisms were *S. aureus* (55.3%) and *P. aeruginosa* (41.7%). Other less prevalent bacterial isolates include coagulase-negative staphylococci, *Escherichia coli*, *klebsiella spp.*, *Enterobacter spp.*, and *Achromobacter xylosoxidans*. The highest rate of resistance for *S. aureus* was observed to azithromycin and erythromycin (80%), ciprofloxacin (52.3%), clindamycin (44.6%) and tetracycline (43%). Twenty percent of *S. aureus* isolates were methicillin-resistant *S. aureus* (MRSA) and 47.6% were MDR *S. aureus*. For *P. aeruginosa* isolates the highest resistance was to cefepime (38.3%) and levofloxacin (33.3%) and 20% showed MDR phenotype.

**Conclusion:**

Our study demonstrated a significant decline in the prevalence of *P. aeruginosa* infections in comparison to previous studies. We found *S. aureus* to be more prevalent in younger patients, whereas mucoid *P. aeruginosa* showed a shift in prevalence toward older ages. Molecular typing methods showed great diversity between isolates.

## 1. Introduction

Cystic fibrosis (CF) is an inherited genetic disorder which affects 70,000 to 100,000 people around the world [1, 2]. It is caused by a mutation in the transmembrane conductance regulator (CFTR) gene [3]. A defective CFTR results in thickened and viscous mucus secretions in the respiratory tract that cannot be easily cleared by the mucociliary clearance system [1, 4]. The accumulated mucus creates an appropriate niche for bacterial colonization and the development of persistent pulmonary bacterial infections [2, 5]. This malicious cycle of chronic infection, inflammation, and tissue destruction results in a progressive decline in lung function, which is the primary cause of morbidity and mortality in patients with CF [6]. In addition to chronic lung infections, CF patients also experience recurrent episodes of acute decline in pulmonary function called “pulmonary exacerbation” (PE). The general presentations of a common PE are increased cough, change in sputum, shortness of breath, fever, decreased appetite, weight loss, and decrease in spirometric parameters [7]. PEs are often associated with the acquisition of new organisms, a change in the bacterial density, or the expansion of pre-existing strains [8]. Within the very first months of their life, CF patients develop airway infections. *Staphylococcus aureus* is the most dominant bacteria during childhood, but gradually, Gram-negative bacteria, especially *Pseudomonas aeruginosa*, become more dominant [9]. Other prevalent bacteria recovered by conventional culture techniques that are supposed to play a role in pulmonary infections in CF patients are *Burkholderia cepacia* complex species, *Haemophilus influenzae*, *Stenotrophomonas maltophilia*, and *Achromobacter xylosoxidans* [9, 10]. With the increasing survival of CF patients and more advanced methods for nontuberculous mycobacteria identification, the prevalence of NTM isolation from CF sputum samples is reported more frequently. *Mycobacterium avium* complex and *Mycobacterium abscessus* are the most common isolates [11, 12].

Epidemiological studies by molecular typing methods are of great importance to determining the genetic diversity of the pathogens, determining the prevalent molecular types of the isolated bacteria, tracking the spread of infections, and assessing the potential risk of person-to-person transmission of infection. Staphylococcal protein A typing and repetitive extragenic palindromic element PCR typing methods are among the well-established typing techniques used in the study of molecular evolution and outbreak investigations of *S. aureus* and *P. aeruginosa*, respectively [13–16].

Considering the vital impact of PEs on patient health, better understanding the microbiological signals associated with PEs will help to find new therapeutic strategies or predictive biomarkers to reduce the frequency and/or severity of PEs [17]. Given the importance of microbiological surveillance of CF patients, this study aims to investigate the frequency, antimicrobial susceptibility pattern, and molecular typing of bacterial pathogens isolated from CF patients during the PE phase who were admitted to The Cystic Fibrosis Center at Children's Medical Center University Hospital, Tehran, Iran, during a period of one year.

## 2. Material and Methods

### 2.1. Patients and Medical Records

Between March 2018 and February 2019, all confirmed CF patients (sweat test and clinical presentations) with signs of pulmonary exacerbation according to Goss and Burns criteria [18], referred to the cystic fibrosis center at Children's medical center University Hospital, Tehran, Iran, were included in the study. Patients who had multiple referrals over the sampling period were also included. Spontaneous sputum samples were obtained from patients involved during the study period. The patient was asked to rinse her/his mouth, cough, and expectorate the sputum into a sterile container. Sputum samples with high saliva contamination were excluded. When it was not possible to obtain spontaneous sputum samples, an oropharyngeal (OP) swab was used as a sample.

### 2.2. Ethics Statement

The study was approved by the Ethics Committee of Tehran University of Medical Sciences, Tehran, Iran. (code: IR.TUMS.MEDICINE.REC.1397.192). All participants included in the study and/or parents (in the case of children) were provided with oral consent before enrollment in the study. All sputum specimens were produced voluntarily. All the patients' information was kept confidential.

### 2.3. Bacterial Identification

Homogenized sputum or throat swab samples were inoculated on the following culture media for primary screening: general media (blood agar and chocolate agar) and selective/differential media (MacConkey agar, mannitol salt agar, *Burkholderia cepacia* selective agar (BCSA), and stenotrophomonas maltophilia agar). Chocolate agar plates were incubated in 5% CO_2_ atmosphere. Plates were incubated at 37°C for 48 hours [19]. BCSA plates were incubated for a further 5 days at room temperature. Suspected colonies were isolated and subcultured. The isolates were identified to the species level using standard biochemical methods [20]. For the detection of mycobacteria, sputum samples were processed by the modified Petroff's method [21] and inoculated on LJ (Löwenstein-Jensen) medium. All slopes were observed for any signs of growth daily for the first week and then at weekly intervals for 8 weeks. The absence of growth at the end of 8 weeks was regarded as a negative culture.

*S. aureus* and *P. aeruginosa* as the two most prevalent pathogens of CF patient's respiratory tract, were investigated in more details to illustrate a more precise picture of their phenotypic and molecular features.

### 2.4. Antimicrobial Susceptibility Testing

Antimicrobial susceptibility tests were performed by Kirby-Bauer's disc diffusion method according to the guidelines of the Clinical and Laboratory Standards Institute (CLSI, 2019) [22]. To determine the susceptibility pattern of *S. aureus* isolates the following antibiotics were tested: cefoxitin (FOX: 30 *μ*g), rifampin (RP: 5 *μ*g), gentamicin (GEN: 10), ciprofloxacin (CIP: 5), TMP/SMX (TS: 1.25/23.75 *μ*g), clindamycin (C: 2 *μ*g), linezolid (LZD: 30 *μ*g), tetracycline (T: 30 *μ*g), azithromycin (ATH: 15 *μ*g) and erythromycin (E: 15 *μ*g). For *P. aeruginosa* isolates the following antibiotics were tested: imipenem (IMI: 10 *μ*g), ceftazidime (CAZ: 30 *μ*g), meropenem (MEM: 10 *μ*g), piperacillin-tazobactam (PTZ: 100/10 *μ*g), cefepime (CPM: 30 *μ*g), aztreonam (ATM: 30 *μ*g), gentamicin (GM: 10 *μ*g), tobramycin (TN: 10 *μ*g), amikacin (AK: 30 *μ*g), ciprofloxacin (CIP: 5 *μ*g), and levofloxacin (LEV: 5 *μ*g). All the antibiotic discs were purchased from MAST Company (Mast Group Ltd, UK). *E. coli* ATCC 25922 was used as the control strain [23]. Detection of methicillin-resistant* Staphylococcus aureus* (MRSA) isolates was performed by 30 *μ*g cefoxitin antibiotic discs (Mast Group Ltd, UK) according to recommendations of CLSI 2019 [22]. The molecular confirmation of MRSA was accomplished by amplification of the *mec*A gene. Resistance to at least one agent in three or more antimicrobial classes was considered as multidrug resistance (MDR) [24].

### 2.5. Biofilm Formation Assay for *S.aureus* and *P. aeruginosa* Isolates

Biofilm formation was evaluated according to the method of O'Toole and Kolter [25] with some modifications. Briefly, 100 *μ*L of each bacterial suspension, adjusted to McFarland standard 0.5 was inoculated into each well of flat-bottomed 96-well polystyrene microtiter plate (SPL Plastic Labware, Korea). After overnight incubation at 37°C the medium was removed and washed twice with 0.9% NaCl. The formed biofilms were fixed by methanol and stained with 1% (w/v) crystal violet solution; then 33% glacial acetic acid (Merck, Germany) was added to the wells. After 10 minutes the absorbance of solubilized crystal violet was measured at 550 nm. The experiments were performed in triplicate. Uninoculated medium was considered a negative control sample. According to the results of microtiter plate test, biofilm producer isolates were characterized as follows based on the optical density: nonbiofilm producers (OD test < ODc), weak biofilm producers (ODc < OD < 2 × ODc), moderate biofilm producers (2 × ODc < OD < 4 × ODc), and strong biofilm producers (4 × ODc < OD) [26].

### 2.6. Spa Typing for *S. aureus* Isolates

The Spa typing was performed according to Harmsen et al. [27]. Briefly, the polymorphic X region of the *spa* gene was amplified by specific primers. The PCR reaction was performed in 12.5 *μ*L final volumes containing 5 *μ*L of PCR Master Mix (BioFACT, South Korea), 4.5 *μ*L of distilled water, 0.25 *μ*L of each primer, and 2.5 *μ*L of extracted DNA. The PCR amplification conditions were as follows: the initial denaturation at 94°C for five minutes, and the next 35 cycles consisting of a denaturation step at 94°C for 30 seconds, annealing at 58°C for 30 seconds, extension at 72°C for 30 seconds, and a final extension step at 72°C for ten minutes. The *spa* gene PCR products were sequenced at Pishgam Biotech Company (Tehran, Iran). Isolates were assigned to particular spa types according to the guidelines described by the spa typing website (https://www.spaserver.ridom.de) [23].

### 2.7. Repetitive Extragenic Palindromic Element PCR (Rep-PCR) Genotyping for *P.aeruginosa* Isolates

The molecular typing of *P. aeruginosa* isolates was performed by rep-PCR as previously described with some modifications [16]. DNA amplification was performed in a final volume of 25 *μ*l containing 16 *μ*l of 2X Multi-Star PCR Master Mix (BioFACT, South Korea), 1 *μ*l of each primer (rep-F: 5′-ICGICTTATCIGGCCTAC-3′ and rep-R: 5′-IIIICGICGICATCIGGC-3′), 5 *μ*l of distilled water, and 2 *μ*l of the template DNA. The cycling conditions were as follows: initial denaturation for 2 minutes at 95°C, followed by 35 cycles for 1 minute at 95°C, 1 minute at 42°C, 4 minutes at 72°C, and a final extension for 16 minutes at 72°C. The rep-PCR products were loaded on a 1.5% (w/vol) agarose gel and were analyzed by gel electrophoresis at 80 V for 2 h. A 1 kilobase DNA ladder (Thermo Fisher scientific, USA) was used as a molecular size standard. To monitor the reproducibility of the method, a *P. aeruginosa* ATCC 27853 reference strain was used as a control in each PCR reaction. The rep-PCR fingerprints of *P. aeruginosa* isolates were analyzed using GelCompar II software, version 4.0 (Applied Maths, Belgium), on the basis of the number and weight of band differences. The relatedness among isolates was deduced as previously described: linked isolates (similarity above 95%) and different (similarity less than 95%) [28].

### 2.8. Statistical Analysis

Data were analyzed in SPSS software (version 23; IBM Corp, USA). Nominal variables have been described with frequencies and percentages. Continuous variables were described as the mean ± SD. In addition, a binary logistic regression analysis was utilized to establish the association between the outcome variable (presence or absence of different bacteria) and the explanatory variable (age).

A *P* value less than 0.05 was considered statistically significant.

## 3. Results

### 3.1. Study Population

In this descriptive cross-sectional study, 103 samples from 85 CF patients between 8 months and 30 years of age with pulmonary exacerbation signs were collected. Nine patients had two referrals, and four patients had three referrals over the sampling period that were eligible for inclusion in our study. A summary of basic patient demographics and types of samples included in the study are provided in Table 1. Most cases (28.2%) were in age group of 2 to 5 years and the least cases (5.9%) were in 20 to 30 years age group. The most common symptoms observed in patients were increased coughing (74.7%) and reduced FEV1 (59.2%).

### 3.2. Prevalence of Microbial Isolates

A total of 120 bacteria and 9 yeasts were isolated from 103 respiratory samples. The most prevalent isolated species were *S. aureus* and *P. aeruginosa*. Other less prevalent bacterial isolates include coagulase-negative staphylococci, *Escherichia coli*, *klebsiella spp.*, *Enterobacter spp.*, and *Achromobacter xylosoxidans*. In 14.5% (15/103) of the samples, no pathogenic bacteria were isolated. The distribution pattern of each species within different age groups is presented in Table 2.

**Figure 1:**
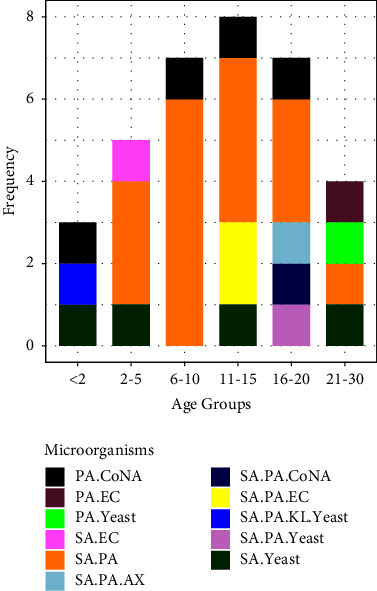
Distribution of coinfection patterns within different age groups. SA, *Staphylococcus aureus*; PA, *Pseudomonas aeruginosa*; KL, *klebsiella spp*; EC, *Escherichia coli*; E.spp, *Enterobacter spp*; CoNA, coagulase negative staphylococci; AX, *Achromobacter xylosoxidans.*

The prevalence of mono-microbial and polymicrobial infections was 52.4% (54/103) and 33.0% (34/103), respectively. *S. aureus* and *P. aeruginosa* coinfections were detected in 22.3% (23/103) of the samples that were mostly from patients in the 6 to 10 age group. Other double, triple, and quadruple coinfection patterns that were detected from patients are presented by age group in Figure 1.

### 3.3. Antibiotic Susceptibility, Biofilm Formation, and Typing

#### 3.3.1. Staphylococcus aureus

**Figure 2:**
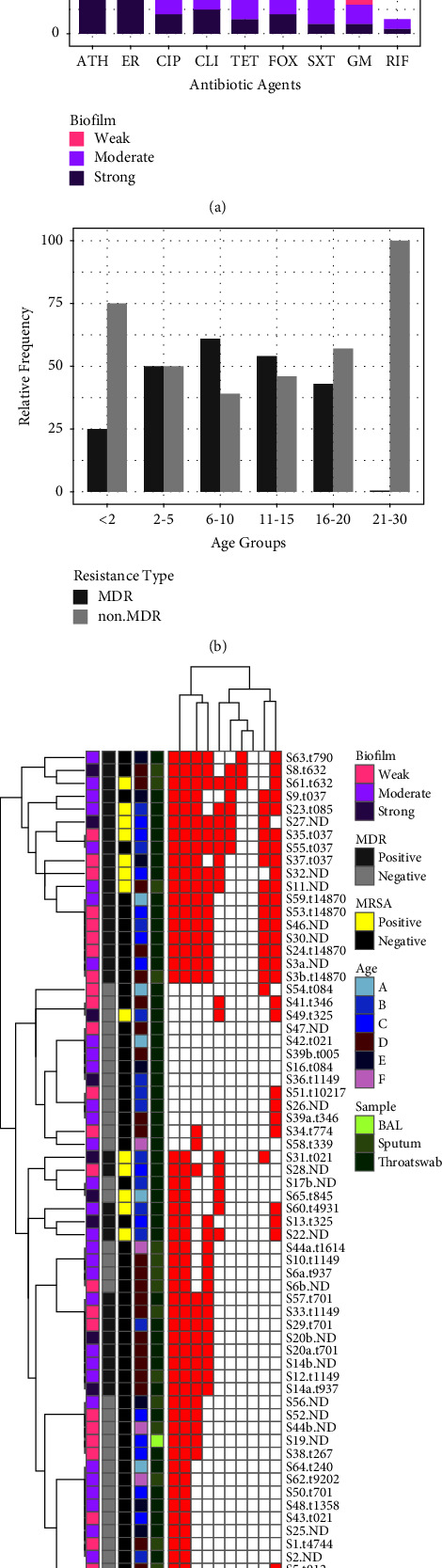
Population structure of *S. aureus* isolates. (a) Relative frequency of resistance to antibiotics in *S. aureus* isolates and biofilm formation status. (b) Relative frequency of MDR and non-MDR*S. aureus* isolates vs. age group. (c) Overview of antimicrobial-resistance profiles and other characteristics of *S. aureus* isolates. ATH, azithromycin; ER, erythromycin; FOX, cefoxitin; RIF, rifampin; GM, gentamicin; CIP, ciprofloxacin; SXT, trimethoprim-sulfamethoxazole; CLI, clindamycin; TET, tetracycline; age group is represented as A < 2, B 2–5, C 6–10, D 11–15, E 16–20, F 20–30 years of age; spa types are shown in front of isolate code; S, *S. aureus*; a and b letters represent the different morphotypes of the same bacteria isolated from the same patient; red squares are representative of resistance and white squares represent susceptibility to the tested antibiotic.

The most prevalent pathogenic bacterial species was *S. aureus* 55.3% (57/103). Seven patients (patient numbers 11, 12 in both two referrals, 26, 28, 53, and 60) were infected with more than one phenotype of *S. aureus* with either different antibiotic susceptibility pattern and/or different colony morphology and biofilm formation status (supplementary Table 1). Thus, in total, 65 morphotypes of *S. aureus* were assessed for antibiotic susceptibility, biofilm formation status, and molecular typing. Of the 65 *S. aureus* isolates tested for susceptibility to 10 antibiotics, the highest rate of resistance was observed to azithromycin and erythromycin 80% (52/65) followed by ciprofloxacin 52.3% (34/65), clindamycin 44.6% (29/65), tetracycline 43% (28/65), TMP/SMX 24.6% (16/65) and cefoxitin 24.6% (16/65). The lowest resistance rates were for gentamicin 10.7% (7/65) and rifampin 4.6% (3/65), and no isolate was resistant to linezolid (Figures 2(a) and 2(c)). Among the 16 cefoxitin-resistant* S. aureus*, 13 isolates were found to be positive for the *mec*A gene and none of them were found to carry the *mec*C gene. The overall prevalence of MRSA and MDR *S. aureus* was 20% (13/65) and 47.6% (31/65), respectively. Four out of 13 of the patients that were positive for MRSA had a *P. aeruginosa* coinfection, one for the *E. coli* and two for the yeast. The highest and lowest prevalences of MDR *S. aureus* were observed in 6 to 10 and 21 to 30 age groups, respectively. The distribution of MDR and non-MDR strains in different age groups is depicted in Figure 2(b). A history of antibiotic use at the time of sampling in patients with MDR *S. aureus* can be found in Table 2 of the supplementary files.

Results of the microtiter plate assay for biofilm production of *S. aureus* isolates showed that all 65 *S. aureus* isolates were biofilm producers in which 8 isolates (12.3%) were strong biofilm producers, 32 (49.2%) moderate, and 25 (38.4%) were weak biofilm producers. The biofilm formation status of resistant strains is illustrated in Figure 2(a). No relation between biofilm formation status and antibiotic resistance was evident.

As presented in Figure 2(c), *S. aureus* isolates fall into three clusters according to their respective resistance patterns. Isolates from the first and third clusters demonstrate similarities in the relative frequencies of resistance combinations and types of samples from which they have been isolated. MRSA isolates mostly fit into first and third cluster and MDR isolates mostly grouped into the first and to a lesser extent to the third cluster. The second cluster mostly included isolates with low relative frequencies of resistance most of them were methicillin susceptible and were mostly isolated from throat swabs.

*Spa* typing of *S. aureus* isolates revealed that the isolates came from a variety of genotypes and are not from a CF-specific clade. While most *spa* types were unique, t037 was found in 4 patients (all were MDR and grouped in the first cluster), t701 (all were MDR/MSSA and grouped in the second cluster), and t021 were found in 3 patients and t1149, t14870, t325, and t084 in 2 patients. The detailed result for each isolate is shown in Figure 2(c) and supplementary Table 1.

#### 3.3.2. Pseudomonas aeruginosa

**Figure 3:**
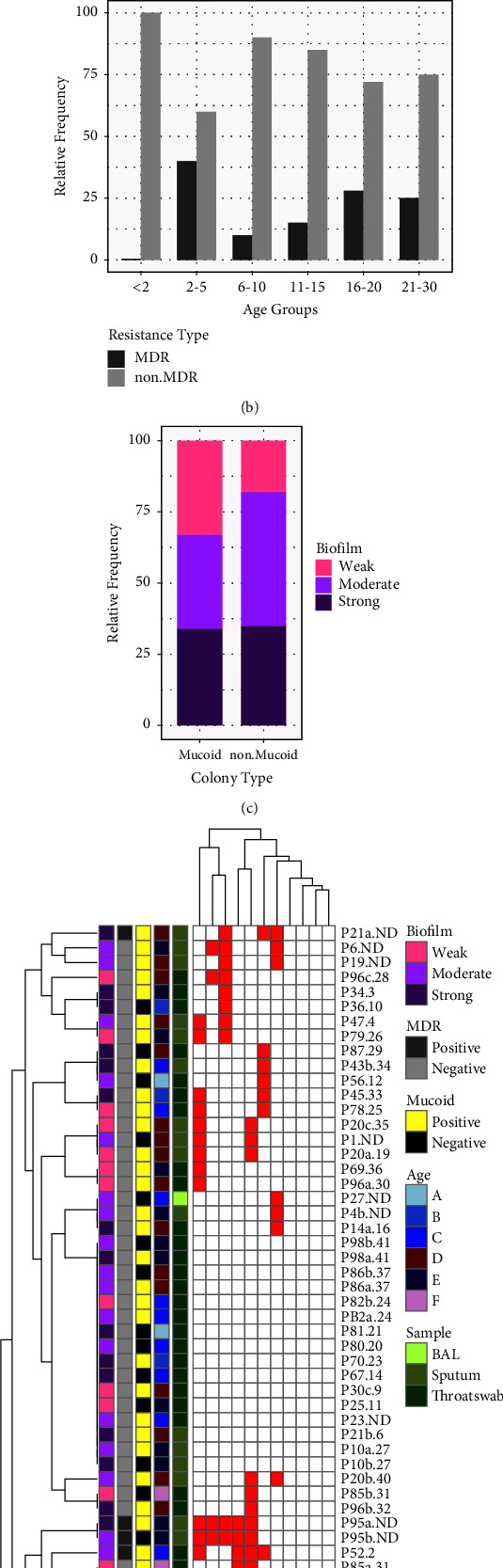
Population structure of *P. aeruginosa* isolates. (a) Relative frequency of resistance to antibiotics in *P. aeruginosa* isolates and biofilm formation status. (b) Relative frequency of MDR and non-MDR*P. aeruginosa* isolates vs. age group. (c) Relative frequency of mucoid/non mucoid phenotype of *P. aeruginosa* and biofilm formation status. (d) Overview of antimicrobial-resistance profiles and other characteristics of *P. aeruginosa* isolates. GM, gentamicin; CIP, ciprofloxacin; IMI, imipenem; CAZ, ceftazidime; MEM, meropenem; PTZ, piperacillin-tazobactam; CPM, cefepime; ATM, aztreonam; TN, tobramycin; AK, amikacin; LEV, levofloxacin; age group is represented as A < 2, B 2–5, C 6–10, D 11–15, E 16–20, F 20–30 years of age; (P) *P. aeruginosa*; a, b and c letters represent the different morphotypes of the same bacteria isolated from the same patient; red squares are representative of resistance and white squares represent susceptibility to the tested antibiotic.

The second most prevalent species was *P. aeruginosa* which was positive in 41.7% (43/103) of the samples. There was a significant positive correlation between increased patients age and *P. aeruginosa* infection (*p* > 0.00); however, we failed to find any correlation between age and other bacteria. Among the *P. aeruginosa* isolates, 71.6% (43/60) showed a mucoid phenotype and were mostly isolated from patients older than 16 years of age (Figures 3(c) and 3(d)). Fourteen patients (patient numbers 4, 6, 10, 14, 19, 20, 23, 30, 43, 85, 86, 96, and 98) were infected with two or three morphotypes of *P. aeruginosa* with a difference in being mucoid/nonmucoid, pigment production, and/or different antibiotic susceptibility pattern (supplementary Table 3). Accordingly, 60 morphotypes of *P. aeruginosa* were evaluated for further investigations.

The highest resistance rate was for cefepime 38.3% (23/60) and levofloxacin 33.3% (20/60), and the lowest resistance was for ceftazidime 6.6% (4/60), imipenem 5.0% (3/60), and tobramycin 3.3% (2/60) (Figure 3(a)). Prevalence of MDR *P. aeruginosa* was 20% (12/60) which is represented by age group in Figure 3(b). The highest and lowest prevalences of MDR *P. aeruginosa* were observed in the 2 to 5 and less than two age groups, respectively. Additional data on antibiotic consumption at the time of sampling in patients with MDR *P. aeruginosa* can be found in Table 4 of the supplementary files.

A microtiter plate assay for *P. aeruginosa* isolates demonstrated that 59/60 (98.3%) were biofilm producers, from which 23 (39.0%) produced strong biofilm, 19 (32.2%) moderate biofilm, and 17 (28.8%) weak biofilms. The biofilm formation status in resistant strains and mucoid/nonmucoid isolates is illustrated in Figures 3(a) and 3(c) respectively.

Using a similarity cut-off of 95%, rep-PCR typing allowed the differentiation of 53 *P. aeruginosa* isolates into 41 types (supplementary Figure 1). Rep-types are presented in numbers for each isolate in Figure 3(d) and supplementary Table 3.

As it is evident in Figure 3(d), *P. aeruginosa* isolates fall into four clusters. Isolates within the first cluster show a high level of resistance to amikacin, are more likely to express a mucoid phenotype, and are isolated mostly from adolescent patients. Isolates in the second cluster showed the lowest individual and resistance combination levels of all populations. They are mostly isolated from throat swabs, and the relative prevalence of the mucoid phenotype is the lowest in this group. A relatively high level of resistance is apparent in the third and fourth cluster. MDR *P. aeruginosa* isolates with a prevalence of 20% are grouped in these two clusters.

## 4. Discussion

Cystic fibrosis patients are predisposed to bacterial colonization and infections throughout their life. This study provides an overview of the bacterial profile and their antibiotic resistance pattern from the CF patients visiting the main referral children hospital in Iran. The most prevalent isolate in our study was *S. aureus* 55.3% (57/103) which is roughly comparable with Ukraine (40.8%) [29], but more than studies conducted in India (15.7%) [30], Canada (24%) [31], and Iran on 2012 (9.3%) [32] and Germany (63.3%) [33]. In the latest report from Iran on 2021, the prevalence of *S. aureus* was 15.6% [34]. This inconsistency may be due to dissimilar demographic composition, different identification methods, and longer incubation times in our study which improve the chance of slow-growing strains isolation. In 2008, 50.9% of CF patients included in the cystic fibrosis foundation (CFF) patient registry had positive *S. aureus* cultures and the most affected groups were children between 6 to 10 and adolescents of 11 to 17 years of age which is identical to our study [35, 36]. Several reports have suggested that chronic MRSA infection is associated with a high decline rate of lung function, failure to recover lung function after a pulmonary exacerbation and decreased survival [37, 38]. The prevalence of MRSA in our study was 20% which is similar to Chmiel et al. who reported the prevalence of MRSA infection at 25% in the USA, as compared to 11% in Canada and Europe [39]. MRSA infections have been increasingly reported among populations with CF worldwide [40]. According to the annual reports of CFF Patient Registry on 2020, the highest prevalence of MRSA occurs in individuals between the ages of 10 and 30, whereas MSSA peaks among those younger than 15. The pattern was different in our study, in which MSSA was predominant in 11 to 15 age group and MRSA peaked in younger patients, mostly in 2 to 5-year-old patients (Figure 2(c)). The high frequency of MRSA in younger children in our study may probably be caused by a circulating MRSA clone in the community. Another possible explanation may be the fact that in countries where care protocols have improved, MRSA colonization has shifted toward older ages which reflects the critical role of health care policies and appropriate treatment in improving the CF population health.

The results of *spa* typing showed a great diversity across *S. aureus* isolates infecting individuals with CF (Figure 2(c) and supplementary Table 1). In this study, spa t037 was the most frequent lineage, which is different from results reported from the USA and Argentina, where spa t002 was more prevalent in pediatric CF patients [41, 42]. To the best of our knowledge, there is no information on the pattern of common *spa* types of *S. aureus* isolates from cystic fibrosis patients in Iran, and this is the first report. Based on the analysis, we conclude that the *S. aureus* clinical isolates surveyed here are not from clonal lineages that transmit between CF patients but instead are from multiple lineages. The majority of patients that were coinfected with two or more phenotypically different *S. aureus* isolates, harbored the same *spa* type except for patient numbers 11 and 53 that were colonized with two different *spa* types (supplementary Table 1). Patient number 13 had two visits with an interval of 8 month in both of which *S. aureus* t632 was isolated that may imply the stability of initial strain (supplementary Table 1).

The second-most prevalent isolate was *P. aeruginosa* 41.7% (43/103). With more rapid detection and development of antipseudomonal antibiotics among the pediatric population, new *P. aeruginosa* acquisitions can be more effectively eradicated [43]. Annual report of the CFF Patient Registry on 2020 demonstrated the percentage of individuals with a positive culture for *P. aeruginosa* has continued to decline over time, with the largest decrease observed among individuals younger than 18 years (44.5 percent had a positive culture in 2000 compared with 18.1 percent in 2020) [44, 45]. In our study the prevalence varied significantly by age, from 4.8% in patients under 2 years of age to 30.4% in 16 to 20-year-old patients (*p* > 0.00). In a study, conducted at Mofid Children's Hospital in Iran from 2004 to 2010, the main infecting pathogen was *P. aeruginosa* (38.8%) [32] which is not identical to our results. A recent study from Iran reported *P. aeruginosa* as the most common bacterial isolate from CF patients with a prevalence of 55.5% [34]. The most important underlying reason for this discrepancy may be the time of sampling and the different population age composition.

As shown in Figure 3(d), the overall resistance rate is low among *P. aeruginosa* isolates. Isolates within the first cluster which show a high level of resistance to amikacin are mostly isolated from adolescent patients. Higher amikacin resistance in this group in comparison to others may have been caused by a higher treatment frequency with this antibiotic for chronic infections with *P. aeruginosa*. A relative high level of resistance is apparent in the third and fourth cluster, where there are more adults that are likely to have been received more antibiotics throughout their life span. In our study, ceftazidime and imipenem were the most effective antibiotics against *P. aeruginosa* isolates, and the least effective antibiotics were cefepime and levofloxacin (Figure 3(a) and supplementary Table 3). A study in Iran showed the most effective antibiotics against *P. aeruginosa* isolated from CF patients included rifampin, vancomycin, imipenem, ciprofloxacin, ofloxacin, and ceftazidime and the less effective antibiotics were penicillin, ampicillin, cephalothin, and cefixime, respectively [32]. The latest report from Iran on *P. aeruginosa* isolates from CF patients showed the highest resistance rate was observed for gentamicin, followed by amikacin, imipenem, and ceftazidime, and the lowest resistance rates were observed in piperacillin-tazobactam [34].

A multi-center study conducted in the United Kingdom, Belgium, and Germany on the antimicrobial susceptibility of *P. aeruginosa* isolates showed high resistance levels of 54% for penicillins (ticarcillin, piperacillin, and piperacillin-tazobactam), 59% for ceftazidime, 46% for amikacin, 27% for ciprofloxacin, 20% for carbapenems, and 16% for tobramycin. Resistance levels were notably much higher than that which have been reported before [46–48]. High levels of resistance were mainly attributed to the epidemic clone the Liverpool Epidemic Strain (LES) which was prevalent in the UK, and four MDR sequence type 958 (ST958) isolates that were found to be spread over the three countries [49]. Differing antibiotic treatment strategies, demographics, genetic characteristics of the population, laboratory methods, and hygiene practice may justify the difference observed between studies.

By rep-PCR typing, *P. aeruginosa* isolates were divided into 41 types with a 95% similarity cut-off level (supplementary Figure 1) [28]. Analysis of rep-PCR results indicate that the majority of our patients are infected with different lineages (Figure 3(d) and supplementary Table 3). The great diversity may be associated with microevolutionary events in the airway environment of CF patients [50]. The surveillance of the different morphotypes of *P. aeruginosa* isolated from individual CF patients to recognize when new morphotypes that may be more resistant to antimicrobial agents emerge is of great concern. As for patients' number 6, 7, 10, and 14, who had multiple referrals, different morphotypes with a higher resistance rate were isolated (supplementary Table 3).

*S. aureus* and *P. aeruginosa* are the two most commonly recognized bacterial pathogens associated with chronic lung infections in patients with CF [51]. A number of studies have shown that coinfection is associated with diminished lung function and more rapid pulmonary decline [2, 17, 52]. In our study, 22.3% (23/103) of the cases were coinfected with *S. aureus* and *P. aeruginosa* (Figure 1). The global rates of *S. aureus*/*P. aeruginosa* coinfection around the world range from 8.6% to 60% with an average of 28.3% which is highest for patients in their mid-twenties [41].

Almost all *P. aeruginosa* and *S. aureus* (more than 98%) were biofilm producers (Figures 2(a), 2(c), 3(a), 3(c) and 3(d)) which exemplify the important role of biofilm mode of growth as a key factor facilitating persistence of infection in the CF respiratory tract. A report from Iran showed that 76% and 67% of *P. aeruginosa* and *S. aureus* isolates from CF patients were biofilm producers, respectively [26]. As it is evident in Figure 3(c), contrary to expectations, nonmucoid showed a greater ability to form more strong biofilm structures than mucoid isolates. This may be due to the inadequacy of the microtiter plate method for measuring the ability of *P. aeruginosa* from CF patients to form biofilms. As previously mentioned, *P. aeruginosa* strains in a CF lung are more likely to form aggregate structures known as microcolonies by connecting to each other and to the mucin rather than attaching to a surface [53].

In spite of significant advances in treatment procedures focusing on CFTR potentiator drugs, respiratory infections remain an important cause of lung disease [39]. Thus, monitoring of such infections and the changing trend of infectious agents that may play a role in pulmonary exacerbation is vitally important in CF patients. The limitations of our study are the small number of patients from a single CF center and the unavailability of clinical histories and previous culture results. Lack of corroboration of culture methods with molecular approaches is another limitation which could help to have a better scheme of the bacterial profile during exacerbation. Furthermore, anaerobic bacteria, viruses, and fungi which may play a crucial role in proceeding exacerbation of respiratory parameters were not assessed in our study.

## 5. Conclusion

This study provides a picture of the bacterial profile from the respiratory tract of CF patients.

*S. aureus* and *P. aeruginosa* were the most common isolated bacteria during pulmonary exacerbation episodes. Our study demonstrated a significant decline in the prevalence of *P. aeruginosa* infections in comparison to previous studies. We found *S. aureus* to be more prevalent in younger patients, whereas mucoid *P. aeruginosa* was the dominant species in adults.

The polymicrobial nature of airway infections in CF patients makes it problematic to isolate fastidious and slow-growing microorganisms such as *H. influenzae* and *B. cepacia* complex, which may be hampered by more dominant species. It is suggested to use molecular techniques to detect these less frequent and fastidious organisms. The current study highlights the importance of epidemiological surveillance of CF pulmonary exacerbations. Early detection and prompt eradication treatment at the very onset of infection may effectively prevent the establishment of chronic infection and reduce the risk of morbidity, mortality, recurrent pulmonary exacerbation, and hospitalization.

## Figures and Tables

**Table 1:** Patients' demographic data and clinical presentations.

Patients (*N* = 85)	Number (%)
Mean age^*∗*^	9.41 ± 6.33
*Age range*	
<2	8 (9.5)
2–5	24 (28.2)
6–10	22 (25.9)
11–15	15 (17.6)
16–20	11 (12.9)
21–30	5 (5.9)
*Gender*	
Male	40 (46.6)
Female	45 (53.4)
Number of samples	103
*Types of samples*	
Sputum	22 (21.4)
Throat swab	80 (77.7)
BAL	1 (1.0)
*Signs of pulmonary exacerbation*	
Fever (oral temperature > 38°)	2 (1.9)
More frequent coughing	77 (74.7)
Increased sputum volume	55 (53.3)
Loss of appetite	27 (26.2)
Weight loss of at least 1 kg	36 (34.9)
Symptoms of upper respiratory tract infection	56 (54.3)
Decreased FEV1%	61 (59.2)
Nail clubbing	26 (25.2)

^*∗*^presented as mean ± SD.

**Table 2:** Distribution of microorganisms in different age groups.

Microorganisms	Age groups (years) (number)/number (%)						
	<2 (15)	2–5 (24)	6–10 (24)	11–15 (32)	16–20 (25)	21–30 (9)	Total number = 129
*Staphylococcus aureus*	5 (8.7)	14 (24.6)	12 (21.2)	16 (28.1)	7 (12.2)	3 (5.2)	57 (55.3)
*Pseudomonas aeruginosa*	2 (4.8)	5 (11.8)	9 (20.4)	11 (25.7)	13 (30.4)	3 (6.9)	43 (41.7)
Coagulase negative staphylococci	3 (25.0)	3 (25.0)	2 (16.7)	1 (8.3)	3 (25.0)	0	12 (11.6)
Yeast	3 (33.4)	0	1 (11.1)	2 (22.2)	1 (11.1)	2 (22.2)	9 (8.7)
*Escherichia coli*	0	1 (25.0)	0	2 (50.0)	0	1 (25.0)	4 (3.8)
*Klebsiella spp.*	2 (100.0)	0	0	0	0	0	2 (1.9)
*Enterobacter spp.*	0	1 (100.0)	0	0	0	0	1 (0.9)
*Achromobacter xylosoxidans*	0	0	0	0	1 (100.0)	0	1 (0.9)

## Data Availability

The data used to support the findings of this study are included within the article and the supplementary information files.

## Supplementary Materials

Supplementary Materials The following supporting information can be downloaded. Table S1: characteristics of *Staphylococcus aureus* isolates; Table S2: history of antibiotic usage by patients with MDR *S. aureus*. Table S3: characteristics of *Pseudomonas aeruginosa* isolates. Table S4: history of antibiotic usage by patients with MDR *P. aeruginosa*. Figure S1: rep-PCR dendrogram of *P. aeruginosa* isolates.
