# Supplementary material for: Distribution and Characteristics of Bacteria Isolated from Cystic Fibrosis Patients with Pulmonary Exacerbation
Source: Can J Infect Dis Med Microbiol. 2022 Dec 24;2022:5831139. doi: 10.1155/2022/5831139 (PMC9805393; doi:10.1155/2022/5831139)
Supplement: Supplementary Materials — The following supporting information can be downloaded. Table S1: characteristics of Staphylococcus aureus isolates; Table S2: history of antibiotic usage by patients with MDR S. aureus. Table S3: characteristics of Pseudomonas aeruginosa isolates. Table S4: history of antibiotic usage by patients with MDR P. aeruginosa. Figure S1: rep-PCR dendrogram of P. aeruginosa isolates. [file 5831139.f1.zip › Revised Supplementary data.docx]

**Supplementary data**

Table 1. Characteristics of *Staphylococcus aureus* isolates

| **Patients** | **Isolates** | **Date of isolation** | **Antibiotic resistance pattern******* | **Biofilm formation†** | **spa typing** |
| --- | --- | --- | --- | --- | --- |
| P1 | P1-Sa | Apr 24,2018 | AZ, ER | M | t4744 |
|  |  |  |  |  |  |
| P2 | P2-Sa | Apr 24,2019 | AZ, ER | M | ND^§^ |
|  |  |  |  |  |  |
| P7  P7 | P7-Sa1  P7-Sa2 | Apr 24,2018  Nov 29,2018 | CIP, SXT, CLI, TET, AZ, ER  CIP, SXT, CLI, TET, AZ, ER | M  W | ND  ND |
|  |  |  |  |  |  |
| P11    P11 | P11-Sa1  P11-Sa2  P11-Sa3 | May 17,2018    Aug 23,2018 | CIP, SXT, CLI, TET, AZ, ER  TET, AZ, ER  CIP, SXT, CLI, TET, AZ, ER | W  W  M | t14870  t012  t14870 |
|  |  |  |  |  |  |
| P12    P12 | P12-Sa1  P12-Sa2  P12-Sa3  P12-Sa4 | May 24,2018    Jul 26,2018 | CLI, AZ, ER  CLI, AZ, ER  CIP, CLI, AZ, ER  CIP, CLI, AZ, ER | M  W  S  M | ND  t937  t937  ND |
|  |  |  |  |  |  |
| P13  P13 | P13-Sa1  P13-Sa2 | Jun 21,2018  Feb 14,2019 | RIF, GM, CIP, CLI, TET, AZ, ER  FOX, RIF, GM, CIP, CLI, TET, AZ, ER | S  M | t632  t632 |
|  |  |  |  |  |  |
| P15 | P15-Sa | Jul 5,2018 | GM, CIP, SXT, TET, AZ, ER | M | t037 |
|  |  |  |  |  |  |
| P19  P19  P19 | P19-Sa1  P19-Sa2  P19-Sa3 | Jul 5,2018  Jul 12,2018  Oct 25,2018 | CLI, AZ, ER  CIP, CLI, AZ, ER  CIP, CLI, AZ, ER | M  M  W | t1149  t1149  t1149 |
|  |  |  |  |  |  |
| P20 | P20-Sa | Jul 12, 2018 | FOX, CIP, SXT, CLI, TET, AZ, ER | M | ND |
|  |  |  |  |  |  |
| P22 | P22-Sa | Jun 26,2018 | CLI, TET, AZ, ER | S | t325 |
|  |  |  |  |  |  |
| P25 | P25-Sa | Aug 2, 2018 | - | M | t084 |
|  |  |  |  |  |  |
| P26 | P26-Sa1 | Aug 2, 2018 | TET, AZ, ER | M | ND |
|  | P26-Sa2 |  | FOX, AZ, ER | M | ND |
|  |  |  |  |  |  |
| P27 | P27-Sa | Aug 2, 2018 | CIP, AZ, ER | M | ND |
|  |  |  |  |  |  |
| P28    P28 | P28-Sa1  P28-Sa2  P28-Sa3 | Aug 9, 2018    Jan 24, 2018 | CIP, CLI, AZ, ER  CIP, CLI, AZ, ER  CIP, CLI, AZ, ER | W  M  M | t701  ND  t701 |
|  |  |  |  |  |  |
| P31 | P31-Sa | Aug 23,2018 | FOX, CLI, TET, AZ, ER | S | ND |
|  |  |  |  |  |  |
| P32 | P32-Sa | Aug 23, 2018 | FOX, GM, CIP, SXT, TET, AZ, ER | M | t085 |
|  |  |  |  |  |  |
| P34  P34 | P34-Sa1  P34-Sa2 | Sep 6, 2018  Dec 13, 2018 | AZ, ER  AZ, ER | W  W | ND  t1358 |
|  |  |  |  |  |  |
| P36 | P36-Sa | Sep 6, 2018 | TET | M | ND |
|  |  |  |  |  |  |
| P38 | P38-Sa | Oct 4, 2018 | FOX, GM, CIP, SXT, AZ, ER | M | ND |
|  |  |  |  |  |  |
| P39 | P39-Sa | Oct 4, 2018 | FOX, CIP, AZ, ER | S | ND |
|  |  |  |  |  |  |
| P40 | P40-Sa | Oct 4, 2018 | CIP, CLI, AZ, ER | W | t701 |
|  |  |  |  |  |  |
| P41 | P41-Sa | Oct 4, 2018 | CIP, SXT, CLI, TET, AZ, ER | W | ND |
|  |  |  |  |  |  |
| P42 | P42-Sa | Oct 25, 2018 | FOX, SXT, AZ, ER | W | t021 |
|  |  |  |  |  |  |
| P46 | P46-Sa | Oct 11, 2018 | FOX, CIP, CLI, TET, AZ, ER | S | ND |
|  |  |  |  |  |  |
| P48 | P48-Sa | Oct 11, 2018 | CIP, TET | W | t774 |
|  |  |  |  |  |  |
| P49 | P49-Sa | Oct 11, 2018 | FOX, GM, CIP, SXT, CLI, TET, AZ, ER | W | t037 |
|  |  |  |  |  |  |
| P50 | P50-Sa | Nov 1, 2018 | - | W | t1149 |
|  |  |  |  |  |  |
| P51 | P51-Sa | Nov 1, 2018 | FOX, CIP, SXT, TET, AZ, ER | W | t037 |
|  |  |  |  |  |  |
| P52 | P52-Sa | Nov 1, 2018 | CIP, AZ, ER | W | t267 |
|  |  |  |  |  |  |
| P53 | P53-Sa1  P53-Sa2  P53-Sa3 | Nov 1, 2018 | TET  -  FOX, TET | W  M  M | t346  t005  t346 |
|  |  |  |  |  |  |
|  |  |  |  |  |  |
| P54 | P54-Sa | Nov 11, 2018 | - | W | t021 |
|  |  |  |  |  |  |
| P58 | P58-Sa | Nov 22, 2018 | AZ, ER | M | t021 |
|  |  |  |  |  |  |
| P60 | P60-Sa1  P60-Sa2 | Nov 22, 2018 | CLI, AZ, ER  CIP, AZ, ER | W  M | ND  t1614 |
|  |  |  |  |  |  |
|  |  |  |  |  |  |
| P66 | P66-Sa | Dec 6, 2018 | - | W | ND |
|  |  |  |  |  |  |
| P70 | P70-Sa | Dec 13, 2018 | FOX, TET | M | t325 |
|  |  |  |  |  |  |
| P71 | P71-Sa | Dec 13, 2018 | AZ, ER | S | t701 |
|  |  |  |  |  |  |
| P74 | P74-Sa | Dec 20, 2018 | TET | M | t10217 |
|  |  |  |  |  |  |
| P78 | P78-Sa | Dec 27, 2018 | CIP, AZ, ER | W | ND |
|  |  |  |  |  |  |
| P80 | P80-Sa | Jan 3, 2019 | CIP, SXT, CLI, AZ, ER | W | t14870 |
|  |  |  |  |  |  |
| P81 | P81-Sa | Jan 3, 2019 | SXT | W | t084 |
|  |  |  |  |  |  |
| P83 | P83-Sa | Jan 10, 2019 | FOX, GM, CIP, SXT, CLI, TET, AZ, ER | W | t037 |
|  |  |  |  |  |  |
| P84 | P84-Sa | Jan 10, 2019 | CIP, AZ, ER | M | ND |
|  |  |  |  |  |  |
| P88 | P88-Sa | Jan 24, 2019 | CIP | M | t339 |
|  |  |  |  |  |  |
| P89 | P89-Sa | Jan 31, 2019 | CIP, SXT, CLI, TET, AZ, ER | M | t14870 |
|  |  |  |  |  |  |
| P90 | P90-Sa | Jan 31, 2019 | FOX, TET, AZ, ER | M | t4931 |
|  |  |  |  |  |  |
| P94 | P94-Sa | Feb 14, 2019 | AZ, ER | M | t9202 |
|  |  |  |  |  |  |
| P97 | P97-Sa | Feb 21, 2019 | RIF, CIP, CLI, TET, AZ, ER | M | t790 |
|  |  |  |  |  |  |
| P102 | P102-Sa | Feb 28, 2019 | AZ, ER | M | t240 |
|  |  |  |  |  |  |
| P103 | P103-Sa | Feb 28, 2019 | FOX, AZ, ER | S | t845 |

* AZ, azithromycin; ER, erythromycin; FOX, cefoxitin; RIF, rifampin; GM, gentamicin; CIP, ciprofloxacin; SXT, trimethoprim-sulfamethoxazole; CLI, clindamycin; TET, tetracycline, † W, weak; M, moderate; S, strong, § ND, not determined

Table 2. History of antibiotic usage by patients with MDR *S. aureus*

| **Patient code** | **Isolate code** | **Age group†** | **Antibiotic usage at the time of sampling** | **Antibiotic regimen*** | **Duration of antibiotic usage** |
| --- | --- | --- | --- | --- | --- |
| P7 | 7-1 | C | No | - | - |
| P11 | 11-1 | D | No | - | - |
| P13 | 13-2 | D | No | - | - |
| P15 | 15-4 | E | No | - | - |
| P20 | 20-5 | D | Yes | AZ, LEV | 2 Years |
| P21 | 21-6 | D | Yes | AZ, AC | 1 Week |
| P22 | 22-1 | C | Yes | Not known | Not known |
| P24 | 24-1 | D | Yes | AZ, CIP | 2 Months |
| P24 | 24-2 | D | Yes | AZ, CIP | 2 Months |
| P28 | 28-1 | D | No | - | - |
| P28 | 28-2 | D | No | - | - |
| P31 | 31-3 | B | Yes | AZ | 3 Months |
| P32 | 32-4 | B | Yes | Not known | Not known |
| P33 | 33-3 | D | No | - | - |
| P38 | 38-2 | C | Yes | AZ, AK | 1 Month |
| P39 | 39-1 | C | Yes | AZ | 1 Month |
| P40 | 40-1 | B | Yes | AZ, AK | 3 Months |
| P41 | 41-1 | C | No | - | - |
| P42 | 42-1 | B | Yes | AZ | 3 Weeks |
| P46 | 46-2 | C | No | - | - |
| P47 | 47-3 | D | No | - | - |
| P49 | 49-1 | C | No | - | - |
| P51 | 51-2 | E | No | - | - |
| P62 | 62-2 | B | No | - | - |
| P80 | 80-3 | C | Not known | - | - |
| P83 | 83-1 | B | Yes | AZ, AK | 1 Year |
| P87 | 87-2 | D | Not known | - | - |
| P89 | 89-2 | A | No | - | - |
| P90 | 90-3 | B | Yes | AZ, CIP | 4 Months |
| P91 | 91-3 | D | Yes | AZ, AK, AC, LEV | 2 Weeks |
| P97 | 97-3 | E | Yes | AZ, AK, LEV | 1 Month |

**†** Age group is represented as A <2, B 2-5, C 6-10, D 11-15, E 16-20 years of age

* AZ, azithromycin; CIP, ciprofloxacin; AK, amikacin; AC, Amoxicillin Clavulanate; LEV, levofloxacin

Table 3. Characteristics of *Pseudomonas aeruginosa* isolates

| **Patients** | **Isolates** | **Date of isolation** | **Antibiotic resistance pattern** | **Biofilm formation****†** | **Mucoid/Non mucoid** | **Rep-PCR Type** |
| --- | --- | --- | --- | --- | --- | --- |
| P1 | P1-Pa | Apr 24,2018 | CPM, LEV | M | Non mucoid | ND^§^ |
|  |  |  |  |  |  |  |
| P4 | P4-Pa1  P4-Pa2 | Apr 24,2018 | CAZ, MEM, PTZ  MEM | M  M | Mucoid  Mucoid | ND  ND |
|  |  |  |  |  |  |  |
| P6  P6  P6 | P6-Pa1  P6-Pa2  P6-Pa3  P6-Pa4 | Apr 24,2018  Dec 27, 2018  Feb 14, 2019 | MEM, GM, AK  CPM, AK  CPM, GM, AK, CIP, LEV  CPM, GM, AK, CIP, LEV | M  W  S  M | Mucoid  Mucoid  Mucoid  Non mucoid | ND  26  ND  ND |
|  |  |  |  |  |  |  |
| P7  P7 | P7-Pa1  P7-Pa2 | Apr 24,2018  Nov 29, 2018 | MEM, PTZ, CPM, ATM, LEV  CAZ, MEM, PTZ, CPM, ATM, LEV | M  S | Non mucoid  Non mucoid | ND  15 |
|  |  |  |  |  |  |  |
| P10  P10  P10 | P10-Pa1  P10-Pa2  P10-Pa3  P10-Pa4 | May 17,2018  Sep 6, 2018  Dec 13, 2018 | -  -  AK  CPM | M  S  S  W | Mucoid  Non mucoid  Mucoid  Mucoid | 27  27  3  36 |
|  |  |  |  |  |  |  |
| P14 | P14-Pa1  P14-Pa2 | Jun 28, 2018 | MEM  IMI, MEM, CIP, LEV | S  S | Mucoid  Mucoid | 16  17 |
|  |  |  |  |  |  |  |
|  |  |  |  |  |  |  |
| P15 | P15-Pa1 | Jul 5,2018 | CIP, LEV | S | Mucoid | 8 |
|  | P15-Pa2 |  | CIP, LEV | W | Mucoid | 7 |
|  |  |  |  |  |  |  |
| P19  P19  P19 | P19-Pa1  P19-Pa2  P19-Pa3  P19-Pa4 | Jul 5,2018  Jul 12,2018  Oct 25,2018 | MEM, AK  MEM, ATM, AK  -  CPM, AK | M  S  S  M | Mucoid  Mucoid  Mucoid  Mucoid | ND  ND  6  4 |
| P20 | P20-Pa1 | Jul 12, 2018 | CPM, LEV | W | Mucoid | 19 |
|  | P20-Pa2 |  | MEM, LEV | M | Mucoid | 40 |
|  | P20-Pa3 |  | CPM, LEV | W | Mucoid | 35 |
|  |  |  |  |  |  |  |
| P23  P23 | P23-Pa1  P23-Pa2  P23-Pa3 | Jul 26,2018  Jan 10, 2019 | -  -  - | M  M  NP | Mucoid  Mucoid  Mucoid | ND  24  24 |
|  |  |  |  |  |  |  |
|  |  |  |  |  |  |  |
| P25 | P25-Pa | Aug 2, 2018 | - | W | Non mucoid | 11 |
|  |  |  |  |  |  |  |
| P27 | P27-Pa | Aug 2, 2018 | MEM | M | Non mucoid | ND |
|  |  |  |  |  |  |  |
| P30 | P30-Pa1  P30-Pa2  P30-Pa3 | Aug 23,2018 | PTZ, CPM, GM, TN, AK  IMI, PTZ, CPM, GM, AK, LEV  - | W  W  W | Mucoid  Mucoid  Mucoid | 5  5  9 |
|  |  |  |  |  |  |  |
|  |  |  |  |  |  |  |
|  |  |  |  |  |  |  |
| P36 | P36-Pa | Sep 6, 2018 | AK | S | Non mucoid | 10 |
|  |  |  |  |  |  |  |
| P38 | P38-Pa | Oct 4, 2018 | CPM, GM, AK | M | Mucoid | 1 |
|  |  |  |  |  |  |  |
| P43 | P43-Pa1 | Oct 25, 2018 | CPM, GM | S | Mucoid | 38 |
|  | P43-Pa2 |  | ATM | S | Mucoid | 34 |
|  |  |  |  |  |  |  |
| P44 | P44-Pa | Oct 25, 2018 | IMI, CPM, AK, CIP | W | Mucoid | 33 |
|  |  |  |  |  |  |  |
| P45 | P45-Pa | Oct 11, 2018 | CPM, ATM | S | Mucoid | 33 |
|  |  |  |  |  |  |  |
| P52 | P52-Pa | Nov 1, 2018 | CPM, ATM, CIP, LEV | M | Mucoid | 2 |
|  |  |  |  |  |  |  |
| P56 | P56-Pa | Nov 22, 2018 | ATM | M | Non mucoid | 12 |
|  |  |  |  |  |  |  |
| P61 | P61-Pa | Nov 29, 2018 | CIP, LEV | S | Mucoid | 13 |
|  |  |  |  |  |  |  |
| P67 | P67-Pa | Dec 6, 2018 | - | S | Non mucoid | 14 |
|  |  |  |  |  |  |  |
| P70 | P70-Pa | Dec 13, 2018 | - | S | Mucoid | 23 |
|  |  |  |  |  |  |  |
| P77 | P77-Pa | Dec 27, 2018 | CPM, CIP, LEV | S | Mucoid | 22 |
|  |  |  |  |  |  |  |
| P78 | P78-Pa | Dec 27, 2018 | CPM, ATM | W | Mucoid | 25 |
|  |  |  |  |  |  |  |
| P80 | P80-Pa | Jan 3, 2019 | - | M | Non mucoid | 20 |
|  |  |  |  |  |  |  |
| P81 | P81-Pa | Jan 3, 2019 | - | S | Non mucoid | 21 |
|  |  |  |  |  |  |  |
| P84 | P84-Pa | Jan 10, 2019 | CAZ,MEM,CPM,ATM,GM,TN,AK,CIP,LEV | M | Mucoid | 39 |
|  |  |  |  |  |  |  |
| P85 | P85-Pa1  P85-Pa2 | Jan 17, 2019 | CIP, LEV  LEV | W  W | Mucoid  Non mucoid | 31  31 |
|  |  |  |  |  |  |  |
|  |  |  |  |  |  |  |
| P86 | P86-Pa1  P86-Pa2 | Jan 24, 2019 | -  - | M  M | Mucoid  Non mucoid | 37  37 |
|  |  |  |  |  |  |  |
|  |  |  |  |  |  |  |
| P87 | P87-Pa | Jan 24, 2019 | ATM | S | Non mucoid | 29 |
|  |  |  |  |  |  |  |
| P94 | P94-Pa | Jan 31, 2019 | CPM, CIP, LEV | W | Non mucoid | 18 |
|  |  |  |  |  |  |  |
| P96 | P96-Pa1  P96-Pa2  P96-Pa3 | Feb 21, 2019 | CPM  LEV  GM, AK | W  S  W | Mucoid  Mucoid  Mucoid | 30  32  28 |
|  |  |  |  |  |  |  |
| P97 | P97-Pa | Feb 21, 2019 | CAZ, CPM, ATM, GM, AK | M | Mucoid | ND |
|  |  |  |  |  |  |  |
| P98 | P98-Pa1  P98-Pa2 | Feb 21, 2019 | -  - | S  M | Mucoid  Non mucoid | 41  41 |
|  |  |  |  |  |  |  |

* IMI, imipenem; CAZ, ceftazidime; MEM, meropenem; PTZ, piperacillin-tazobactam; CPM, cefepime; ATM, aztreonam; GM, gentamicin; TN, tobramycin; AK, amikacin; CIP, ciprofloxacin; LEV, levofloxacin, † W, weak; M, moderate; S, strong, § ND, not determined

Table 4. History of antibiotic usage by patients with MDR *P. aeruginosa*

| **Patient code** | **Isolate code** | **Patient age group†** | **Antibiotic usage at the time of sampling** | **Antibiotic regimen*** | **Duration of antibiotic usage** |
| --- | --- | --- | --- | --- | --- |
| P4 | 4-1 | E | No | - | - |
| P7 | 7-2 | B | No | - | - |
| P21 | 21-2 | D | Yes | AZ, AC | 1 Week |
| P30 | 30-1 | D | Yes | AZ, AK, CIP | 1 Year |
| P30 | 30-2 | D | Yes | AZ, AK, CIP | 1 Year |
| P44 | 44-1 | F | Yes | AK, LEV, TS | 1 Month |
| P52 | 52-1 | C | Not known | - | - |
| P62 | 62-1 | B | No | - | - |
| P84 | 84-3 | E | Yes | TS | 3 Months |
| P95 | 95-1 | E | Yes | AZ, CIP, DOX | 2 Months |
| P95 | 95-2 | E | Yes | AZ, CIP, DOX | 3 Months |
| P97 | 97-2 | E | Yes | AZ, AK, LEV | 1 Month |

† Age group is represented as A <2, B 2-5, C 6-10, D 11-15, E 16-20, F 20-30 years of age

*AZ, azithromycin; CIP, ciprofloxacin; AK, amikacin; AC, amoxicillin clavulanate; TS, trimethoprim / sulfamethoxazole; DOX, doxycycline; LEV, levofloxacin
