# Supplementary figures and images for: Distribution and Characteristics of Bacteria Isolated from Cystic Fibrosis Patients with Pulmonary Exacerbation
Source: Can J Infect Dis Med Microbiol. 2022 Dec 24;2022:5831139. doi: 10.1155/2022/5831139 (PMC9805393; doi:10.1155/2022/5831139)

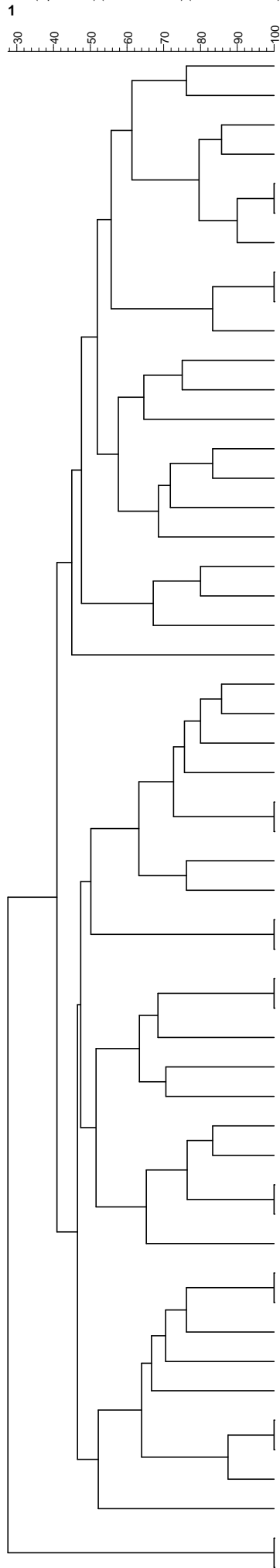

1

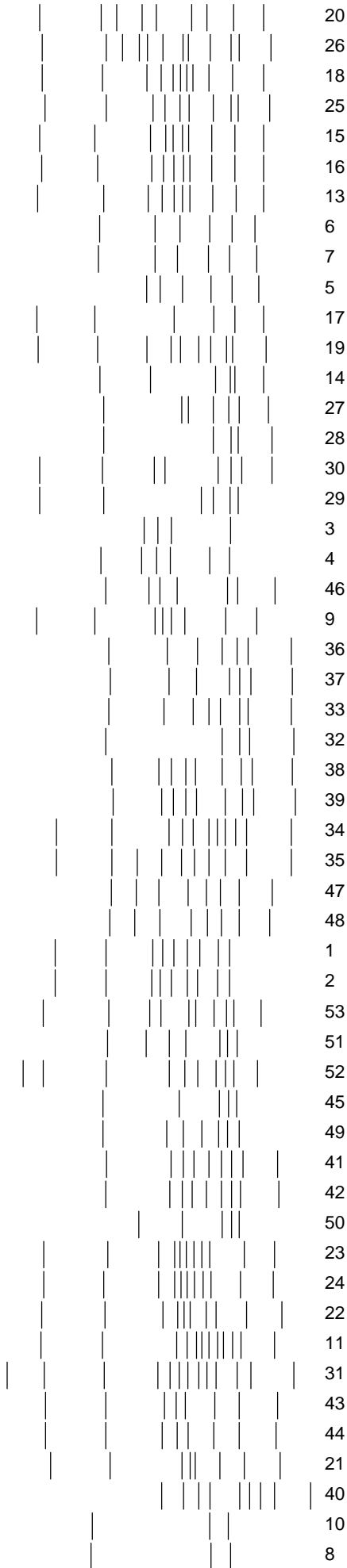

Supplement: Supplementary Materials — The following supporting information can be downloaded. Table S1: characteristics of Staphylococcus aureus isolates; Table S2: history of antibiotic usage by patients with MDR S. aureus. Table S3: characteristics of Pseudomonas aeruginosa isolates. Table S4: history of antibiotic usage by patients with MDR P. aeruginosa. Figure S1: rep-PCR dendrogram of P. aeruginosa isolates. [file 5831139.f1.zip › supplementary fig 1.pdf]
